# Solid-State Reaction in Cu/a-Si Nanolayers: A Comparative Study of STA and Electron Diffraction Data

**DOI:** 10.3390/ma15238457

**Published:** 2022-11-27

**Authors:** Evgeny T. Moiseenko, Vladimir V. Yumashev, Roman R. Altunin, Galina M. Zeer, Nataliya S. Nikolaeva, Oleg V. Belousov, Sergey M. Zharkov

**Affiliations:** 1 Laboratory of Electron Microscopy, Siberian Federal University, 79 Svobodny Ave., 660041 Krasnoyarsk, Russia; 2 Institute of Chemistry and Chemical Technology, Federal Research Center KSC SB RAS, Akademgorodok 50/24, 660036 Krasnoyarsk, Russia; 3 Kirensky Institute of Physics, Federal Research Center KSC SB RAS, Akademgorodok 50/38, 660036 Krasnoyarsk, Russia

**Keywords:** copper silicide, thin films, nanolayer, solid-state reaction, phase formation, kinetics, activation energy, enthalpy, DSC, electron diffraction

## Abstract

The kinetics of the solid-state reaction between nanolayers of polycrystalline copper and amorphous silicon (a-Si) has been studied in a Cu/a-Si thin-film system by the methods of electron diffraction and simultaneous thermal analysis (STA), including the methods of differential scanning calorimetry (DSC) and thermogravimetry (TG). It has been established that, in the solid-state reaction, two phases are formed in a sequence: Cu + Si → η″-Cu_3_Si → γ-Cu_5_Si. It has been shown that the estimated values of the kinetic parameters of the formation processes for the phases η″-Cu_3_Si and γ-Cu_5_Si, obtained using electron diffraction, are in good agreement with those obtained by DSC. The formation enthalpy of the phases η″-Cu_3_Si and γ-Cu_5_Si has been estimated to be: ΔH_η″-Cu3Si_ = −12.4 ± 0.2 kJ/mol; ΔH_γ-Cu5Si_ = −8.4 ± 0.4 kJ/mol. As a result of the model description of the thermo-analytical data, it has been found that the process of solid-state transformations in the Cu/a-Si thin-film system under study is best described by a four-stage kinetic model R3 → R3 → (Cn-X) → (Cn-X). The kinetic parameters of formation of the η″-Cu_3_Si phase are the following: *E_a_* = 199.9 kJ/mol, log(*A*, s^−1^) = 20.5, *n* = 1.7; and for the γ-Cu_5_Si phase: *E_a_* = 149.7 kJ/mol, log(*A*, s^−1^) = 10.4, *n* = 1.3, with the kinetic parameters of formation of the γ-Cu_5_Si phase being determined for the first time.

## 1. Introduction

Cu-Si-based materials have been widely investigated for applications in the technologies of recording information [1], solar power industry [2], and as catalysts for obtaining nanomaterials based on carbon and zinc oxides [3,4]. Cu_3_Si-based nanowire structures are promising for the application as electrical contacts in nanotransistors [5]. Using Cu_5_Si–Si/C-based composite materials has been considered as a method of increasing anode characteristics in ionic lithium batteries [6]. The application of Cu_15_Si_4_ nanowires as a material for anodes in ionic lithium batteries allows increasing their capacity [7].

**Figure 1:**
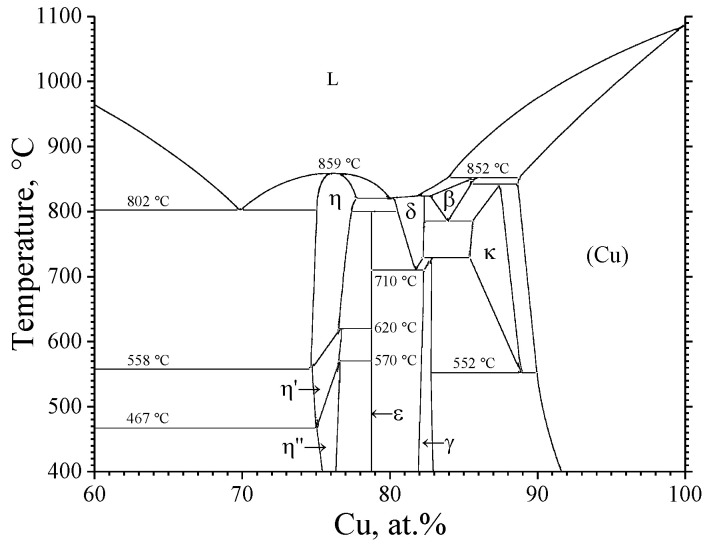
Phase diagram of the Cu-Si system, adopted from [13].

According to the phase diagram (Figure 1), in the Cu-Si system, the formation of the following phases is possible: η-Cu_3_Si, η′-Cu_3_Si, η″-Cu_3_Si; ε-Cu_15_Si_4_, δ-Cu_4_Si, γ-Cu_5_Si, β-Cu_6_Si, κ-Cu_7_Si (Table 1). In this case, the phases η-Cu_3_Si, η′-Cu_3_Si, δ-Cu_4_Si, β-Cu_6_Si, and κ-Cu_7_Si are high-temperature ones (the area of existence is above 467 °C). As is predicted by the model of the effective heat of formation (EHF) [8], phases in a solid-state reaction are to be formed successively as EHF increases. In the case of the Cu-Si system, the formation sequence of the low-temperature phases determined using the data given in [9] is the following: η″-Cu_3_Si → ε-Cu_15_Si_4_ → γ-Cu_5_Si. The experimental results [10,11] confirm that in the solid-state reaction between silicon and copper in thin films, the η″-Cu_3_Si phase is the first one to be formed. However, it is worth noting that the data on the structure of this phase are rather scarce. For example, the most comprehensive description of the structure of the η″-Cu_3_Si phase is presented in [12], where this phase (η″-Cu_3_Si) is said to have the space group P-31c with the lattice parameters a = b = 4.0612 Å, c = 14.6723 Å. The structure of the phases ε-Cu_15_Si_4_ and γ-Cu_5_Si is well known: the ε-Cu_15_Si_4_ phase has the space group I-43d with the lattice parameters a = b = c = 9.718 Å [PDF 4+ card #04-014-4307], and the γ-Cu_5_Si phase has the space group P4_1_32 with the lattice parameters a = b = c = 6.222 Å [PDF 4+ card #00-004-0841].

Of interest is the kinetics of phase formation in the solid-state reaction between copper and silicon nanolayers. Most investigations of the kinetics of phase formation in the solid-state reaction in the Cu-Si system are devoted to the Cu_3_Si phase [10,16,17]. Regarding the formation of the η″-Cu_3_Si phase, various studies present different estimates of the activation energy (from 72 to 268 kJ/mol) [10,16,17,18,19,20]. Moreover, an insufficient amount of experimental data has been obtained for Cu/Si thin films with the bilayer thickness of ~100 nm. For example, in [10] for (Cu/a-Si) multilayer films with the bilayer thickness of 86–160 nm, the growth of the η″-Cu_3_Si phase was found to follow the parabolic law *x*^2^ = *k*^2^*t*, with the activation energy of formation of the η″-Cu_3_Si phase being equal to 94 kJ/mol. For the phases ε-Cu_15_Si_4_ and γ-Cu_5_Si, there are no data on the kinetics of their formation.

The present study describes the results of a complex analysis of solid-state reaction processes between nanolayers of polycrystalline copper and amorphous silicon in bilayer and multilayer Cu/a-Si films. A combination of the methods of differential scanning calorimetry and in situ electron diffraction allowed us to obtain the information on the kinetics and phase formation mechanism for the phases η″-Cu_3_Si and γ-Cu_5_Si, as well as to perform a comparative analysis of the kinetic characteristics obtained by both methods.

## 2. Materials and Methods

Cu/a-Si bilayer and (Cu/a-Si)_30_ multilayer nanofilms were obtained by pulse DC magnetron sputtering. The basic residual pressure was 1 × 10^−4^ Pa, and the argon pressure during sputtering was 0.26 Pa. High-purity materials (Girmet Ltd., Moscow, Russia) Cu (99.997 wt.%) and monocrystalline Si (100) *n*-type (silicon 99.999 wt.%, phosphorus concentration 8 × 10^18^ atom/cm^3^) were used as a target. The film thickness was controlled using an INFICON SQC-310 thin-film deposition controller. The individual layer thickness in the bilayer and multilayer thin films was 70 ± 1 nm for the Cu layer and 25 ± 1 nm for the Si layer. The deposition rate of Cu was 0.43 nm/s and that of Si was 0.17 nm/s. Carbon-coated TEM grids were used for the deposition of the Cu/a-Si bilayer film. Glass substrates covered by a thin layer of Sigma-Aldrich 430,102 Polystyrene (Sigma-Aldrich, Darmstadt, Germany) were used for the (Cu/a-Si)_30_ film deposition.

Simultaneous thermal analysis (STA), including the recording of the mass change (by the method of thermogravimetry (TG)) and heat flow (by the method of differential scanning calorimetry (DSC)) of the (Cu/a-Si)_30_ multilayer nanofilms, was performed using a thermal analyzer Jupiter STA 449C («NETZSCH», Selb, Germany) in a Pt-Rh crucible, with the sample weight being 28.5 ± 0.1 mg. The samples were heated in a dynamic argon atmosphere (99.999% pure) at a flow rate of 100 sccm. The analysis of the (Cu/a-Si)_30_ thin multilayer films was performed upon heating the sample from 40 up to 400 °C at a rate of 5, 10, and 20 °C/min. For the processing of the thermo-analytical data, the software packages NETZSCH Proteus (ver.4.8.4) and NETZSCH Thermokinetics 3 (ver.2006.08) were used. The heat flow calibration of the DSC-TG sensor was performed by measuring the heat capacity of the sapphire disc by the method [DIN 51007:1994-06 Thermal analysis; differential thermal analysis; principles].

The investigations of the microstructure, phase, and elemental composition of the Cu/a-Si bilayer films were carried out using the methods of transmission electron microscopy (TEM), selected area electron diffraction (SAED), and energy-dispersive spectroscopy (EDS) using a transmission electron microscope JEOL JEM-2100 (JEOL, Tokyo, Japan).

The elemental composition of the (Cu/a-Si)_30_ films was studied using a JEOL JSM-7001F scanning electron microscope (JEOL, Tokyo, Japan) equipped with an Oxford Inca Energy 350.

To investigate the process of phase formation during the solid-state reaction between the Cu and a-Si nanolayers, the Cu/a-Si bilayer films were placed onto the Mo TEM grids and heated by a JEOL JEM-2100 heating sample holder. Simultaneously with the heating, SAED patterns were registered, and the temperature of the sample was measured. To study changes in the phase composition of the Cu/a-Si bilayer thin films in the solid-state reaction, the samples were heated from room temperature to 450 °C at different rates: 5, 10, and 15 °C/min. During the heating, electron diffraction patterns were recorded for the film at a rate of 5 frames per minute with the heating rates being 5 and 10 °C/min and at a rate of 7.5 frames per minute with the heating rate being 15 °C/min. The electron diffraction patterns were interpreted using the software DigitalMicrograph (Gatan) (ver.1.85), CrysTBox (ver.1.10) [21,22], and the databases ICDD PDF 4+ [23] and Pearson’s Crystal Data [24]. The analysis of the obtained electron diffraction patterns allowed determining the sequence of phase transformations occurring in the solid-state reaction between dissimilar nanolayers. The authors successfully used the method to investigate the process of phase formation during the solid-state reaction in different thin-film nanosystems: Al/Cu [25,26], Al/Pt [27], Al/Ag [28,29], Cu/Au [30], Al/Fe [31], Fe/Si [32], Fe/Pd [30,33,34], Fe-ZrO_2_ [35], Co-ZrO_2_ [36], and Co-In_2_O_3_ [37]. In the present study, in order to estimate the kinetic parameters of the solid-state reaction (activation energy, pre-exponential factor, and reaction order), a series of heating procedures was performed at different rates.

## 3. Results

### 3.1. Transmission Electron Microscopy and Electron Diffraction Study of the Cu/a-Si Bilayer Films

**Figure 2:**
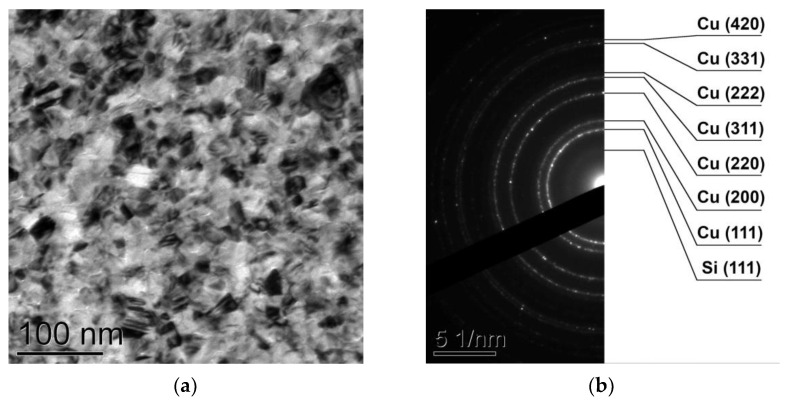
TEM image (**a**) and SAED pattern (**b**) obtained from the Cu/a-Si bilayer thin film at the initial state.

The investigation of the Cu/a-Si bilayer thin films by transmission electron microscopy (Figure 2a) showed that at the initial state the films consisted of copper crystallites with the size of 10–20 nm and amorphous silicon. In the electron diffraction patterns (Figure 2b), one can observe the whole set of diffraction reflections characteristic for the face-centered cubic (FCC) phase of Cu (PDF 4+ card #00-004-0836, space group Fm-3m, a = 3.615 Å), as well as an amorphous halo corresponding to the α-Si phase (PDF 4+ card #00-027-1402, space group Fd-3m, a = 5.431 Å). The investigation by energy-dispersive spectroscopy showed the films to contain Cu = 82.5 ± 0.5 at.%, Si = 17.5 ± 0.5 at.%.

**Figure 3:**
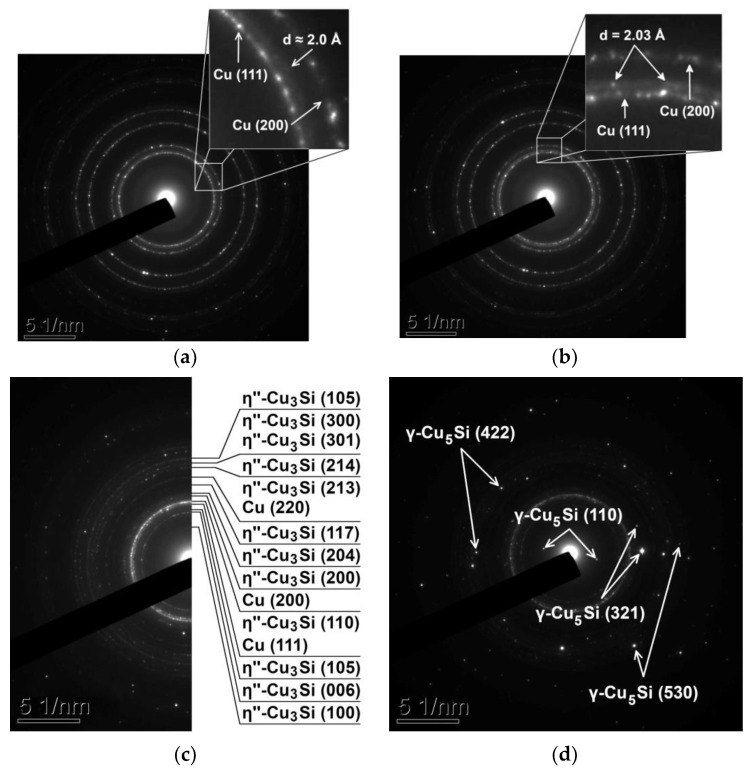
SAED patterns obtained from the Cu/a-Si bilayer thin film at 127 °C (**a**), 166 °C (**b**), 182 °C (**c**), and 255 °C (**d**) during heating at a rate of 5 °C/min.

It was found that upon increasing the heating rate from 5 to 15 °C/min the temperature of the initiation of the solid-state reaction between the copper and silicon nanolayers increased from 128 to 141 °C. The analysis of the diffraction reflections obtained from the Cu/a-Si thin films during heating shows the solid-state reaction, to begin with, and the formation of an amorphous-like intermediate Cu-Si layer with the coherent scattering area ~1 nm, which is accompanied by the appearance of a broadened circular reflection corresponding to the interplanar distance of d ≈ 2.0 Å in the electron diffraction patterns (Figure 3a). Earlier, in [11,18], the solid-state reaction in the Cu/a-Si thin films was shown to begin with the formation of an amorphous-like Cu-Si layer at the interface.

At the following stage of the solid-state reaction, one could observe the beginning of the formation of individual Cu_3_Si nanoclusters with the size of ~1–5 nm, which was accompanied by the appearance of spot reflections in the SAED patterns, with these reflections corresponding to d = 2.03 Å and having low intensity (Figure 3b). The observed reflections d = 2.03 Å are characteristic of the η″-Cu_3_Si phase, whose formation was observed at the next stage of the solid-state reaction, which was accompanied by the formation of polycrystalline circular reflections corresponding to the distances d_110_ = 2.03 Å and d_006_ = 2.45 Å of the η″-Cu_3_Si phase (Figure 3c).

It is worth noting that in the crystal structure databases ICDD PDF 4+ [23] and Pearson’s Crystal Data [24], there are no reliable data on the structure of the η″-Cu_3_Si phase; thus, to identify the reflections of the η″-Cu_3_Si phase, interplanar distances were used which were calculated based on the structural data presented in [12]. At the final stage of the solid-state reaction, one could observe the formation of the γ-Cu_5_Si phase (PDF 4+ card #00-004-0841, space group P4_1_32, a = 6.222 Å), accompanied by the appearance of the reflections that are characteristic of this phase in the electron diffraction patterns (Figure 3d). It is to be emphasized that the formation of the ε-Cu_15_Si_4_ phase was not observed in the framework of this study. According to the phase diagram of the Cu-Si system (see Figure 1), the ε-Cu_15_Si_4_ phase has a very narrow area of homogeneity, and as it is assumed in [38], the ε-Cu_15_Si_4_ phase is thermodynamically stable; however, its formation is impeded, i.e., kinetically inhibited at the nucleation stage.

The initiation temperatures obtained from the analysis of the electron diffraction patterns for each stage of the solid-state reaction (corresponding to the conversion rate *α* ≈ 0.05) in the Cu/a-Si bilayer thin films for different heating rates are presented in Table 2.

**Figure 4:**
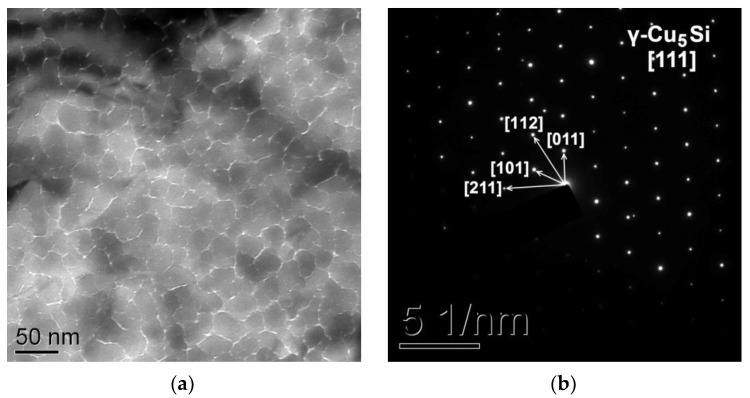
TEM image (**a**) and SAED pattern (**b**) obtained from the Cu/a-Si thin bilayer film after heating to 450 °C.

The analysis of the transmission electron microscopy images (Figure 4a) and electron diffraction patterns (Figure 4b) obtained from the Cu/a-Si bilayer film after heating shows that the film consists of coherently oriented crystallites of the γ-Cu_5_Si phase with the size of 10–30 nm. Thus, the sequence of phases in the solid-state reaction in the Cu/a-Si thin bilayer films is as follows: Cu + Si → η″-Cu_3_Si → γ-Cu_5_Si, which is in agreement with the prediction of the EHF model [8].

Based on the results of the study of the solid-state reaction processes in the Cu/a-Si thin bilayer films by the SAED method, the kinetic parameters of these processes were obtained. To estimate the apparent activation energies and pre-exponential factor, we used the Kissinger–Akahira–Sunose equation [39]:

(1) $$\ln\left(\frac{\beta_{i}}{T_{\alpha ,i}^{2}}\right)=\ln\left(-\frac{AR}{E_{a}}f^{\prime }\left(\alpha \right)\right)-\frac{E_{a}}{RT_{\alpha ,i}},\;$$

where *T_α,i_* is the temperature corresponding to the rate of conversion *α* = 0…1 for the *i*-th measurement (for the classical Kissinger method, *T*_0.5_ = *T_max_*, i.e., the temperatures of the maximum process rate are used for the calculations, corresponding to α ≈ 0.5 for an individual peak).

*β_i_* = *dT*/*dt* is the linear heating rate for the *i*-th measurement.

*E_a_* is the activation energy.

*A* is the pre-exponential factor in the Arrhenius equation.

*R* is the universal gas constant.

*f*’(*α*) = *df*(*α*)/*dα*, where *f*(*α*) is the reaction type according to [40,41]; for the reaction of the first order, *f*(*α*) = (1 − *α*), *f*’(*α*) = −1.

**Figure 5:**
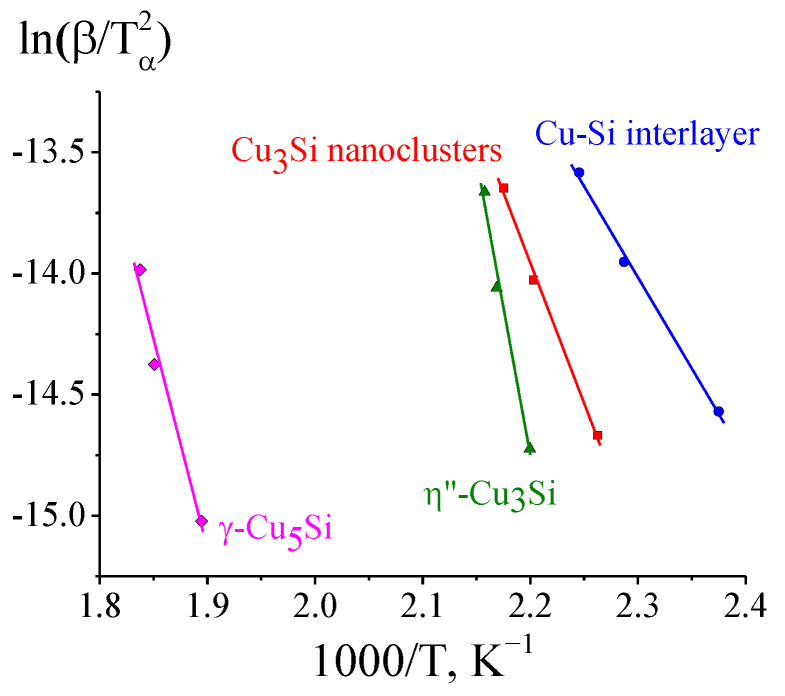
Kissinger plots for the phase formation during the solid-state reaction in the Cu/a-Si bilayer films based on the electron diffraction data.

Here, *T_a_* corresponds to the temperatures of the onset of the reaction (the conversion rate *α* ≈ 0.05) determined through the analysis of the electron diffraction patterns (see Table 2). To estimate the kinetic parameters of different stages of the solid-state reaction in the Cu/a-Si thin bilayer films, we plotted the *ln*(*β*/$T_{\alpha }^{2}$) dependences on 1/*T_a_* (Figure 5). The tangent of the tilt angle in the Kissinger dependences, as well as in the case of the Arrhenius dependence, is determined by the activation energy and corresponds to the *E_a_/R* value. The common logarithm of the pre-exponential factor *A* was calculated from the value corresponding to the intersection with the *Y* axis (Intercept): *log(A)* = [*Intersept* + *ln(E_a_/R)]*/*ln10*. As a result, we obtained the values of the kinetic parameters for different stages of the solid-state reaction (Table 3). A similar method was used in [42] to estimate the kinetic parameters of metal-induced crystallization by the X-ray diffraction data, while in [43], to analyze the kinetics of the formation of silicides (Co, Pt, Ni)-Si, this was carried out by a change in resistivity.

### 3.2. Thermokinetic Analysis of (Cu/a-Si)_30_ Multilayer Films

In order to study the kinetics of the solid-state reaction in the Cu-Si system by simultaneous thermal analysis, (Cu/a-Si)_30_ multilayer films with the thicknesses of individual layers Cu = 70.0 ± 0.5 nm and Si = 25.0 ± 0.5 nm were obtained. The analysis of the multilayer films by the EDS method showed the films to contain 83.0 ± 0.5 at.% Cu and 17.0 ± 0.5 at.% Si.

**Figure 6:**
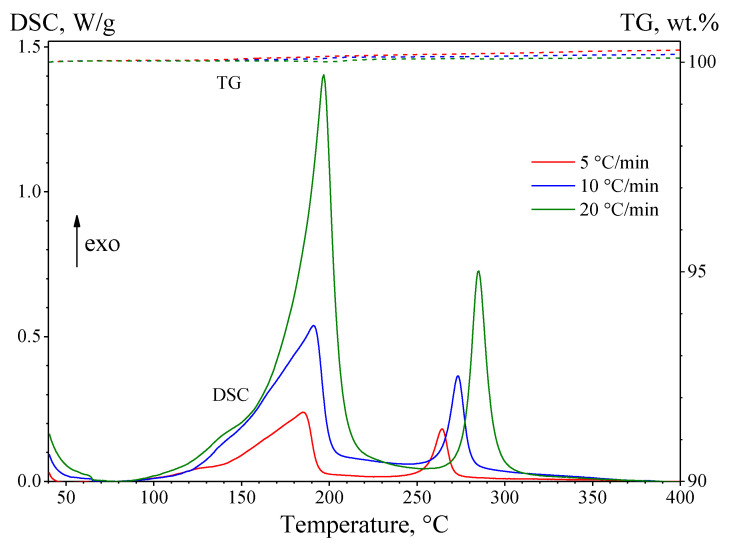
DSC-TG curves of the (Cu/a-Si)_30_ multilayer sample at different heating rates: 5, 10, and 20 °C/min.

As a result, on the DSC curves (Figure 6) in the low-temperature region of 90–230 °C, a poorly resolved exothermic peak was observed, consisting of several components, indicating the multi-stage nature of the processes occurring in this region. In addition, the DSC curves show a high-temperature exothermic peak in the temperature range of 230–320 °C, represented by a single component. The components of the complex DSC peaks correspond to the formation of new phases in the (Cu/a-Si)_30_ thin multilayer films. On the thermogravimetric curves (see Figure 6) in the temperature range of 40–400 °C, there are no significant changes in the weight of the sample, which indicates the absence of traces of the organic substrate (polystyrene), and the thermal effects on the DSC curves should be attributed exclusively to the solid-state interaction in the Cu-Si system.

Table 4 presents the characteristic temperatures of the components of the low-temperature and high-temperature exothermic peaks observed on the DSC curves for different rates of heating the (Cu/a-Si)_30_ thin multilayer films: *T_onset_* is the temperature of the onset of the process, *T_max_* is the temperature corresponding to the maximum rate of the process (i.e., upon the conversion rate *α* ≈ 0.5), and *T_end_* is the temperature of the end of the process:

As a result of the analysis of the DSC curves, the low-temperature exothermic peak was found to consist of three components corresponding to the processes of formation of new phases during the solid-state reaction in the (Cu/a-Si)_30_ thin multilayer films. It should be noted that the first two components of the low-temperature peak on the DSC curve are poorly resolved, which complicates the kinetic analysis of these stages of the solid-state reaction. The comparison of the data obtained by DSC and SAED suggests that the first component of the low-temperature peak on the DSC curves should correspond to the formation of the intermediate Cu-Si layer, the second component should correspond to the formation of the Cu_3_Si nanoclusters, and the third one corresponds to the formation of the η″-Cu_3_Si phase. The high-temperature peak corresponds to the process of the γ-Cu_5_Si phase formation.

The model-free Kissinger and Friedman methods were used to preliminarily estimate the kinetic parameters of the solid-state reaction processes in the thin multilayer (Cu/a-Si)_30_ films. A detailed procedure for analyzing the data obtained by DSC is described in [29].

**Figure 7:**
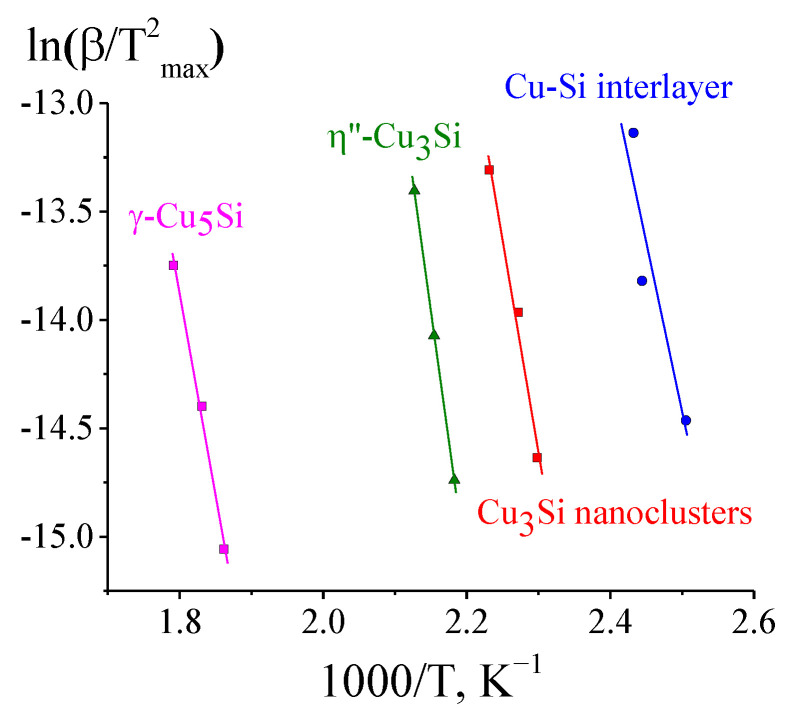
Kissinger plots for the components of the complex DSC peak appearing during the solid-state reaction in the (Cu/a-Si)_30_ multilayer films.

The analysis of the data obtained by DSC (in this case, T_max_ corresponds to the temperatures of the peak maxima on the DSC curve) using the Kissinger method allowed estimating the kinetic parameters for four stages of the solid-state transformation process (Figure 7, Table 5): formation of the intermediate Cu-Si layer, formation of the Cu_3_Si nanoclusters, and formation of the η″-Cu_3_Si and γ-Cu_5_Si phases.

The Friedman’s method is based on the following equation:

(2) $$\ln\left(\beta_{i}{\left(\frac{d\alpha }{dT}\right)}_{\alpha ,\;i}\right)=\ln\left(f\left(\alpha \right)A_{\alpha }\right)-\frac{E_{\alpha }}{RT_{\alpha ,i}},\;$$

where *T_α,i_* is the temperature corresponding to the conversion rate *α* = 0…1 for the *i*-th measurement.

*β_i_* = *dT*/*dt* is the linear heating rate for the *i*-th measurement.

*E_α_* is the activation energy at a conversion rate *α*.

*A_α_* is the pre-exponential factor in the Arrhenius equation at a conversion rate α.

*R* is the universal gas constant.

*f*(*α*) is the reaction type according to [40,41].

**Figure 8:**
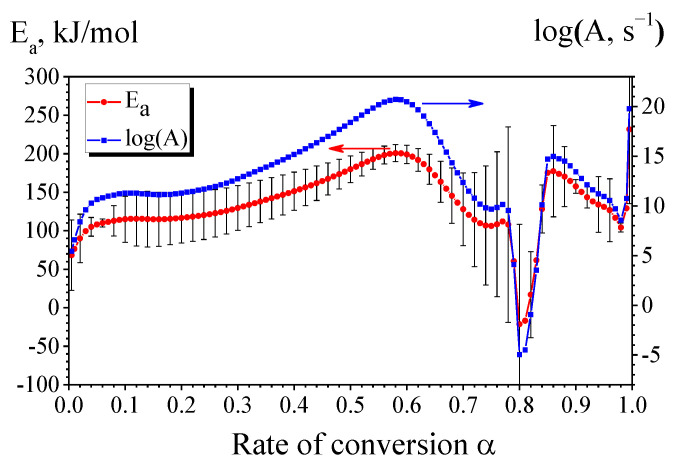
Friedman analysis of the DSC data for (Cu/a-Si)_30_.

The kinetic analysis using the Friedman’s model-free method made it possible to establish that the process of solid-state reaction in the (Cu/a-Si)_30_ multilayer sample allows distinguishing two stages on the curves of dependences of the pre-exponential factor and *E_a_* on the conversion rate (Figure 8): (1) formation of the η″-Cu_3_Si phase with the apparent activation energy *E_a_* = 200 ± 11 kJ/mol and pre-exponential factor log(*A*, s^−1^) ≈ 21 (at *α* = 0.58); (2) formation of the γ-Cu_5_Si phase with *E_a_* = 150 ± 3 kJ/mol and log(*A*, s^−1^) ≈ 13 (at *α* = 0.91). It is difficult to estimate the kinetic parameters of the low-temperature components of the first complex DSC peak using the Friedman method due to the significant effect of diffusion control of the ongoing reaction [44].

Presented below is a comparative table with the estimates of the kinetic parameters of different stages of the solid-state reaction in the Cu/a-Si system, obtained as a result of the analysis of the electron diffraction and DSC data by the Kissinger method, including the analysis of the DSC data by the Friedman method (Table 6).

Due to the fact that the first two components of the low-temperature peak on the DSC curve are poorly resolved, the accuracy of estimating the kinetic parameters of the formation of the intermediate Cu-Si layer and Cu_3_Si nanoclusters by the Kissinger method is low in the case of DSC. At the same time, as can be seen in Table 6, the kinetic parameters of the formation of the η″-Cu_3_Si and γ-Cu_5_Si phases, obtained by the Kissinger method based on the electron diffraction and DSC data, as well as the parameters obtained by the Friedman method from the DSC data, are in good agreement with each other. As regards the comparison with the literature data, different estimates of the activation energy are given in different works for the formation of the η″-Cu_3_Si phase in thin films. For example, in [10], the activation energy for the formation of the η″-Cu_3_Si phase in the (Cu/a-Si)*_n_* multilayer films with a bilayer thickness of 86–160 nm was found to be *E_a_* = 94 kJ/mol. The activation energy for the formation of the η″-Cu_3_Si phase in thin bilayer films (Cu_80nm_/a-Si_120nm_) obtained in [18] was *E_a_* = 72 kJ/mol for the linear growth stage of the phase and *E_a_* = 128 kJ/mol for the parabolic growth stage. In [20], the activation energy E_a_ = 268 kJ/mol was obtained for the formation of the η″-Cu_3_Si phase in Cu/a-Si thin bilayer films with the copper layer thickness of 20 nm and the silicon layer thickness of 5 nm. In addition, in [10], diffusion along the grain boundaries was shown to slow down during the solid-state reaction in the Cu-Si system with a decrease in the size of crystallites. Therefore, rather high values of the activation energy for the formation of the η″-Cu_3_Si phase obtained in this study, *E_a_* = 199–203 kJ/mol, can be accounted for by an increased energy barrier during the Cu diffusion through the amorphous Cu-Si intermediate layer. Here, it should be noted that the kinetic parameters of the γ-Cu_5_Si phase formation were obtained for the first time in this study.

The estimates of the kinetic parameters obtained by the Kissinger method based on the DSC data (see Table 6) were used as initial conditions in the model description of the observed multi-stage process of solid-state transformation by the method of nonlinear multivariate regression [40] using the software Netzsch Thermokinetics 3.

**Figure 9:**
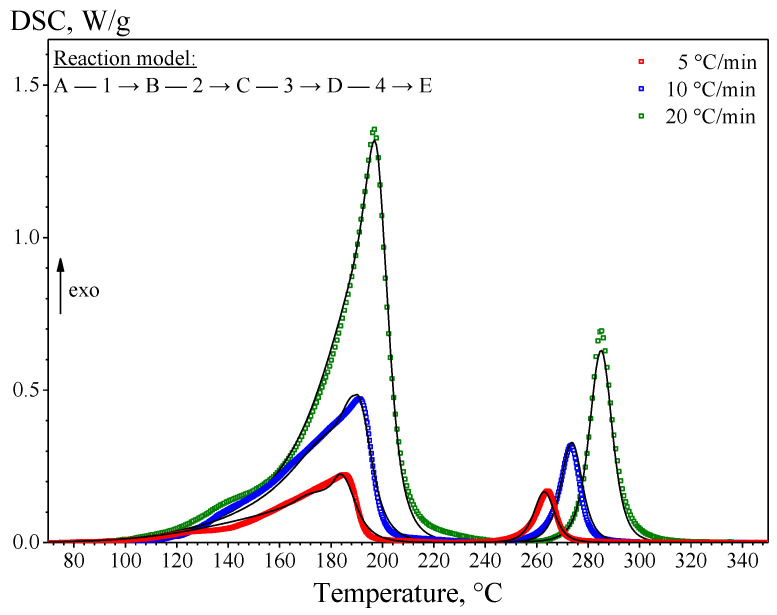
Kinetic multi-curve analysis of the DSC measurements of the (Cu/a-Si)_30_ multilayer films: signs—measured; lines—calculated.

The best modeling results (Table 7) were obtained upon describing the formation stages of the intermediate Cu-Si layer and Cu_3_Si nanoclusters by the equation of the reaction on the three-dimensional phase boundary—R3; and the formation stages of the η″-Cu_3_Si and γ-Cu_5_Si phases are described by the equation of the *n*-th order reaction with Cn-X autocatalysis. A detailed description of the types of reactions is given in [40,41]. The correlation coefficient of the kinetic model obtained by the method of multivariate regression was *R*^2^ = 0.9918 (Figure 9).

**Figure 10:**
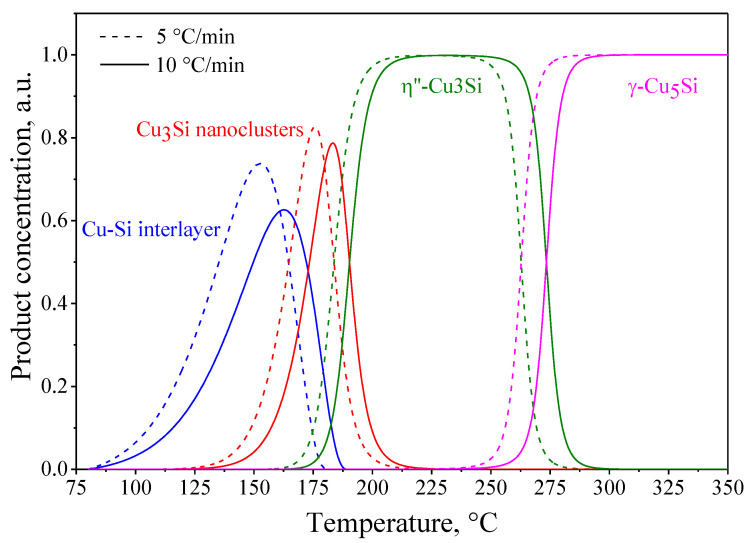
Concentration curves of different stages of the phase formation in the (Cu/a-Si)_30_ thin films calculated using the results of thermokinetic modeling: heating rate of 5 °C/min (dashed lines) and 10 °C/min (solid lines).

Figure 10 shows the dependences of the conventional concentrations of the reaction products in the course of the solid-state reaction in the (Cu/a-Si)_30_ multilayer thin-film system. The dependences were calculated based on the obtained kinetic model R3 → R3 → (Cn-X) → (Cn-X), provided that all the observed processes of phase transformations proceed in a sequence: Cu + Si → (Cu-Si interlayer) → (Cu_3_Si nanoclusters) → η″-Cu_3_Si → γ-Cu_5_Si.

Using the DSC data and the NETZSCH Proteus software package, exothermic effects (per gram of the initial sample) were calculated, corresponding to the low-temperature complex peak and high-temperature peak of the formation of γ-Cu_5_Si (Figure 5), which amounted to 110.7 ± 0.3 J/g and 23 ± 1 J/g, respectively. Based on these calculations, the formation enthalpies of the η″-Cu_3_Si and γ-Cu_5_Si phases were estimated (Table 8).

To estimate the formation enthalpies of the η″-Cu_3_Si and γ-Cu_5_Si phases, the low-temperature peak was decomposed into three components using the NETZSCH Peak Separation software package, according to the procedure described in [45]. The content of the η″-Cu_3_Si and γ-Cu_5_Si phases necessary for the calculation was estimated on the basis of the electron diffraction and EDS data: the maximum content of the η″-Cu_3_Si phase was 65 wt.% of the sample weight, with the content of the γ-Cu_5_Si phase being 95 wt.% of the sample weight. The results of calculation of the formation enthalpies of copper silicide in the (Cu/a-Si)_30_ thin film are presented in Table 8 in comparison with the literature data. One can see that the values of the heat of formation of the η″-Cu_3_Si and γ-Cu_5_Si phases obtained in this study correlate well with the experimental results obtained in [10].

## 4. Conclusions

Using the combination of electron diffraction and simultaneous thermal analysis allowed us to establish that during the solid-state reaction two phases are successively formed between the nanolayers of polycrystalline copper and amorphous silicon (Cu/a-Si): Cu + Si → η″-Cu_3_Si → γ-Cu_5_Si. In this case, the η″-Cu_3_Si phase is formed in three successive stages: formation of an amorphous intermediate layer Cu-Si; formation of Cu_3_Si nanoclusters; and formation of the η″-Cu_3_Si phase.

It is shown that the estimates of the apparent activation energy and pre-exponential factor for the η″-Cu_3_Si and γ-Cu_5_Si phases obtained by analyzing the electron diffraction data using the Kissinger–Akahira–Sunose method are in good agreement with the estimates obtained from the analysis of the DSC data using both the Kissinger method and the Friedman method.

The formation enthalpies of the η″-Cu_3_Si and γ-Cu_5_Si phases were estimated: ΔH_η″-Cu3Si_ = −12.4 ± 0.2 kJ/mol; ΔH_γ-Cu5Si_ = −8.4 ± 0.4 kJ/mol.

The processes of the solid-state reaction in the Cu/a-Si thin-film system were found to be best described by the four-stage kinetic model R3 → R3 → (Cn-X) → (Cn-X), with R3 being the reaction on the three-dimensional phase boundary and (Cn-X) being the *n*-th order reaction with autocatalysis. The kinetic parameters of formation of the η″-Cu_3_Si phase are the following: *E_a_* = 199.9 kJ/mol, log(*A*, s^−1^) = 20.5, *n* = 1.7; and for the γ-Cu_5_Si phase: *E_a_* = 149.7 kJ/mol, log(*A*, s^−1^) = 10.4, *n* = 1.3; with the kinetic parameters of formation of the γ-Cu_5_Si phase being determined for the first time.

## Figures and Tables

**Table 1:** Basic information of the copper silicides.

Phase	Composition, at.% Cu [14]	Temperature Range, °C [15]	Space Group [14]
η-Cu_3_Si	73.7–75.7	558–859	* P-3m1
η′-Cu_3_Si	73.7–75.9	467–620	* P-31m
η″-Cu_3_Si	73.7–77.3	≤570	* P-31c
ε-Cu_15_Si_4_	78.8	≤800	I-43d
δ-Cu_4_Si	81.6–83.1	710–824	P6_3_/mmc
γ-Cu_5_Si	82–83	≤729	P4_1_32
β-Cu_6_Si	83.5–85.7	785–852	Im-3m
κ-Cu_7_Si	85.8–88.4	552–842	P6_3_/mmc

* Correa C.A. et al. [12].

**Table 2:** Characteristic temperatures of the initiation of the phase formation (corresponding to the conversion rate *α* ≈ 0.05) in the Cu/a-Si bilayer film according to the electron diffraction data.

Heating Rate, °C/min	Temperature of the Initiation of Phase Formation, °C			
	Cu-Si Interlayer	Cu_3_Si Nanoclusters	η″-Cu_3_Si	γ-Cu_5_Si
5	148	169	182	255
10	164	181	188	267
15	172	187	190	271

**Table 3:** Kinetic parameters for the phase formation during solid-state reaction in the Cu/a-Si bilayer films based on electron diffraction data.

Step	*E_a_*, kJ/mol	log (*A*, s^−1^)	*R* ^2^
Cu-Si interlayer formation	62 ± 4	5 ± 3	0.9965
Cu_3_Si nanocluster formation	96 ± 6	9 ± 4	0.9959
η″-Cu_3_Si formation	203 ± 24	21 ± 4	0.9859
γ-Cu_5_Si formation	144 ± 23	12 ± 6	0.9752

**Table 4:** Characteristic temperatures of the main exothermic peaks on the DSC curve corresponding to thermochemical transformations in the (Cu/a-Si)_30_ multilayer films.

Heating Rate °C/min	Characteristic Temperatures, °C							
	Peak 1 (Polymodal)					Peak 2 (Monomodal)		
	*T_onset_*	* T_max1_	* T_max2_	*T_max3_*	*T_end_*	*T_onset_*	*T_max_*	*T_end_*
5	104	126	162	185	193	256	264	271
10	123	136	167	191	200	264	273	280
20	118	138	175	197	208	277	285	295

* The position of the hidden peaks was determined from the second derivative of the DSC curve.

**Table 5:** Kinetic parameters for the phase formation during solid-state reaction in the Cu/a-Si bilayer films based on the Kissinger analysis of DSC data.

Step	*E_a_*, kJ/mol	log (*A*, s^−1^)	*R* ^2^
Cu-Si interlayer formation	130 ± 53	15 ± 11	0.8563
Cu_3_Si nanocluster formation	163 ± 21	18 ± 6	0.9831
η″-Cu_3_Si formation	199 ± 2	21 ± 3	0.9999
γ-Cu_5_Si formation	155 ± 12	13 ± 4	0.9942

**Table 6:** Model-free estimation of the kinetic parameters of the solid-state transformation process in the Cu/a-Si thin films obtained using the DSC and electron diffraction data.

Step	Kissinger (ED)		Kissinger (DSC)		Friedman (DSC)	
	*E_a_*, kJ/mol	log(*A*, s^−1^)	*E_a_*, kJ/mol	log(*A*, s^−1^)	*E_a_*, kJ/mol	log(*A*, s^−1^)
Cu-Si interlayer formation	62 ± 4	5 ± 3	130 ± 53	15 ± 11	-	-
Cu_3_Si nanoclusters formation	96 ± 6	9 ± 4	163 ± 21	18 ± 6	-	-
η″-Cu_3_Si formation	203 ± 24	21 ± 4	199 ± 2	21 ± 3	200 ± 11	~21
γ-Cu_5_Si formation	144 ± 23	12 ± 6	155 ± 12	13 ± 4	150 ± 3	~13

**Table 7:** Apparent activation energy, pre-exponential factor, reaction order, and autocatalysis rate constant of each transformation in the (Cu/a-Si)_30_ multilayer films.

Step	Reaction Type	Kinetic Parameters	
1. Cu-Si interlayer formation	R3	log(*A_1_*, s^−1^)	5.4
		*E_a1_*, kJ/mol	65.8
2. Cu3Si nanocluster formation	R3	log(*A_2_*, s^−1^)	14.2
		*E_a2_*, kJ/mol	142.2
3. η″-Cu_3_Si phase formation	Cn-X	log(*A_3_*, s^−1^)	20.5
		*E_a3_*, kJ/mol	199.9
		*n_3_* (reaction order)	1.7
		* log(*K_cat3_*)	0.7
4. γ-Cu_5_Si phase formation	Cn-X	log(*A_4_*, s^−1^)	10.4
		*E_a4_*, kJ/mol	149.7
		*n_4_* (reaction order)	1.3
		* log(*K_cat4_*)	2.8

* Autocatalysis constant for the corresponding reaction.

**Table 8:** Formation enthalpies of copper silicides in the thin films.

Reference	η″-Cu_3_Si, kJ/mol	γ-Cu_5_Si, kJ/mol
Chromic R.R. et al. [10]	−13.6 ± 0.3	−10.5 ± 0.6
This study	−12.4 ± 0.2	−8.4 ± 0.4

## Data Availability

Not applicable.
